# Co-design of Guidance for Patient and Public Involvement in Psychedelic Research

**DOI:** 10.3389/fpsyt.2021.727496

**Published:** 2021-09-30

**Authors:** James B. Close, Julia Bornemann, Maria Piggin, Sandra Jayacodi, Lisa Xiaolu Luan, Robin Carhart-Harris, Meg Jo Spriggs

**Affiliations:** ^1^Centre for Psychedelic Research, Division of Brain Sciences, Imperial College London, London, United Kingdom; ^2^Patient Experience Research Centre, Imperial College London, London, United Kingdom; ^3^Imperial Biomedical Research Centre Public Panel, London, United Kingdom

**Keywords:** psychedelics, research, patient, public, involvement, PPI, guideline, strategy

## Abstract

Within the context of scientific research, patient and public involvement (PPI) is defined as research performed “with” or “by” patients and members of the public, rather than “to,” “about”, or “for” them. When carried out systematically and thoughtfully, PPI has the potential to strengthen the quality and impact of research by fostering accountability, transparency, and relevance. There exist numerous guidelines, frameworks and tools for supporting PPI, however, these do not account for the unique challenges faced in psychedelic research. This paper describes the co-design of guidance intended to help build, evaluate and improve PPI in psychedelic research. A steering group was formed to design and run a co-design workshop alongside public collaborators. Insights from this workshop were analyzed and refined into a comprehensive and readily usable guide for planning PPI specific to the field of psychedelic research. Core values emerging from the process focused on the essential importance of trust, learning, purpose and inclusivity. It is hoped that this guidance will be a starting point for incorporating PPI in future psychedelic research, so that it can grow and adapt as this burgeoning field of research progresses.

## Introduction

Research into “classic” tryptamine psychedelics [e.g., psilocybin, lysergic acid diethylamide (LSD), and dimethyltryptamine (DMT)] is proliferating at a rapid rate. Database searches reveal more than a doubling in the number of peer-reviewed publications relating to psychedelics in the last decade (1). Among this surge is a growing number of clinical and non-clinical trials exploring the action of psychedelics in human subjects (2, 3). Around the globe, research centers choosing to focus on psychedelics shape the manner and direction in which we choose to approach this frontier. Historically, academic research has been carried out with little to no involvement of those outside the core research team, with patients and members of the public seen as a sources of data on which to build and test hypotheses (4). This is changing, rising ethical standards are placing more weight on addressing the inherent power imbalance between researchers as “knowledge creators,” and those who stand to benefit from that knowledge (5).

Within the context of scientific enquiry, patient, and public involvement (PPI) is defined as research performed “with” or “by” patients and members of the public, rather than “to,” “about”, or “for” them (6). Involving patients and public in research is becoming more commonplace, and has risen from a “desirable” to “essential” criteria for many research funding bodies, such as UK Research & Innovation (UKRI), an assembly of six organizations with a combined budget of over £8 billion (7). Furthermore, updated legislation now makes it a duty for healthcare providers to involve patients in the commissioning and decision making process in several governing organizations including the UK's National Institute for Health Research (NIHR) (8), the United Nations Children's Fund (UNICEF) (9), and several Canadian regional health boards (10). Such a collective reprioritization signifies a new gold standard for conducting research, particularly in clinical populations where those with lived experience offer a pragmatic, real world perspective on the development of interventions. Neglecting to recognize the reality of how data will be used downstream can lead to impractical “blue sky” research which ultimately fails to get adopted (11).

Besides a moral and ethical imperative for involving patients and the public in research, a growing evidence base indicates how PPI can strengthen research by improving priority setting (12), recruitment and retention (13, 14), diversity among participants (15, 16), and impact and dissemination of findings (17, 18). Other purported benefits of PPI can be difficult to capture, leaving them open to criticism, for example, a public contributor could gain more agency, or a researcher may improve their ability to explain concepts in plain language (19, 20).

There is limited evidence of PPI taking place in psychedelic studies to date. None of the 16 contemporary clinical trials on classic psychedelics describe PPI activities in their methods or give acknowledgment to public contributors (21–36). While this may not entirely rule out public involvement in these studies, it highlights how reporting is non-essential for the majority of publishers. Notably, a number of these psychedelic studies have come under criticism for a lack of diversity among participants (37, 38), a concern not exclusive to psychedelic research which continues in spite of efforts such as the NIH Revitalization Act (39) and the NIHR INCLUDE project (40, 41). PPI may offer one way to balance inequalities; a study using the psychoactive drug 3,4-Methylenedioxymethamphetamine (MDMA) described PPI methods as a means of improving the relevance of recruitment materials to underrepresented groups (42, 43). Additionally, psychedelic research organizations such as The Chacruna Institute embed PPI principles into their mission statement with the purpose of recognizing the contributions of the traditional healers who have long used naturally occurring psychedelic compounds (44). These are only some examples of the possible benefits of PPI to psychedelic research that remain to be explored.

In spite the growing internal and external incentives for PPI in research, delivery remains a challenge. As a supplementary activity for many, established researchers lack awareness around benefits and methods of PPI (5, 45). Doctoral students often cite restraints on resources or time leading to “tokenistic” or abbreviated attempts at PPI that fail to be recorded in the literature (17). Barriers to PPI in research may result in issues such as stagnant participation rates for people of color, or the overall decline in community engagement over the course of the COVID-19 pandemic (46).

Various tools have been developed to help support PPI in research; a 2019 systematic review identified and classified 64 frameworks (47), these were rarely adopted outside the research groups that developed them, showing how a one-size-fits all approach to PPI guidance may not work. While a number of existing frameworks may be used to help inform PPI in psychedelic research (8, 48–50), none of these account for the unique position which this field of research finds itself, namely: a highly complex socio-political landscape; rapidly changing drug policy; a wealth of knowledge stemming from a long tradition of psychedelic use pre-dating modern research; an underrepresentation of women, Black, Asian, and ethnically diverse populations; the social stigma associated with recreational use; and the 20-year hiatus in psychedelic research. All of these factors are not only important considerations for researchers conducting studies, but also for those taking part, particularly considering the potential for long-term impact on a participant's life (51). In response, the aim of this project was to co-develop guidance for planning PPI in psychedelic research which: (1) is sensitive to the unique challenges faced in psychedelic research, (2) draws on the experience of researchers, those with experience participating in psychedelic trials, and those with experience in PPI, (3) is harmonious with UK standards for PPI, and (4) is rated highly on quality measures for PPI guidance.

The Oxford English Dictionary (OED) defines the word guidance as “help or advice about how to do something.” In an attempt to make the guidance generalizable between both clinical and non-clinical psychedelic trials, we do not aim to provide study specific suggestions. Instead, we hope that guidance will be adaptable and broadly applicable to all types of research conducted in the psychedelic space.

## Methods

A steering group was formed to plan, oversee and review all activities over the course of the production process. Group members comprised of four psychedelic researchers including one clinical academic, a public advisor and a PPI lead. Prior to starting, co-design objectives and methods were circulated, appraised and agreed upon in meetings between group members. Due to existing high quality PPI guidance, it was agreed that a completely original approach would not be necessary or appropriate. For this reason, and to ensure our approach accorded with national standards, we grounded our co-design process in the UK Standards for Public Involvement (8). This is a framework intended for use in public involvement research and is based on six core areas: inclusive opportunities, working together, support and learning, governance, communications, and impact.

### Co-design

For the purpose of this project, co-design was defined as the deliberate involvement of those outside the central research team in the development and evaluation of research improvement initiatives (52). A number of co-design methods have been described, most originating from outside academic settings (53–55). We chose to adapt and supplement an existing method developed by Greenhalgh et al. (47) as this was specifically created for designing custom PPI frameworks, and has been used successfully elsewhere (56, 57). In this method public collaborators and researchers worked together in an interactive participatory approach to systematically capture and build on suggestions.

We identified four distinct collaborator groups as key to bringing a wide breadth of knowledge and experience to discussions. These were: (1) former participants from clinical psychedelic trials; (2) former participants from non-clinical psychedelic trials; (3) PPI experienced public advisors; and (4) researchers from within the field of psychedelics. Collaborators were recruited using a convenience sampling strategy (58), the opportunity was e-mailed to participants of previous psychedelics studies at Imperial College Centre for Psychedelic Research and online via NIHR's People in Research platform (https://www.peopleinresearch.org/). Additional collaborators were found through the Imperial Patient Experience Research Centre (PERC), a core facility of the Imperial Biomedical Research Centre. All collaborators were offered compensation for their time at rates recommended by the NIHR's Payment Guidance for Researchers and Professionals (59). When appropriate, positive action (60) was taken to ensure a diverse age, gender and ethnicity demographic.

**Figure 1:**
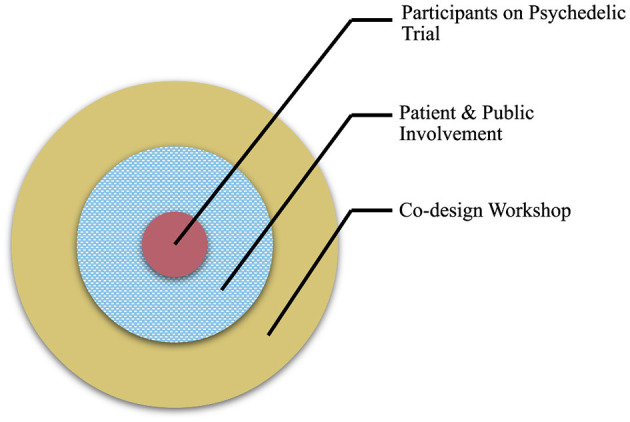
A diagram to illustrate the aim of the co-design workshop. Workshop activities (outer ring) focused on issues related to public contributors and PPI in psychedelic research (middle ring), rather than participants enrolled on a psychedelic study (inner circle).

A facilitated workshop was used to identify and explore salient issues around public involvement in psychedelic research. One month before the workshop collaborators were sent details about the workshop structure and were signposted to information about PPI with the opportunity to ask any questions. Due to COVID-19, there were government imposed restrictions on meeting in groups at the time of the study, and so the workshop was hosted online using a remote platform (Zoom Video Communications, Inc.) and lasted ~2 h. An introduction outlined the aims and structure of the workshop including the important distinction between our focus on PPI for psychedelic trials rather than a focus on participant experience on a psychedelic trial (see Figure 1). Key collaborator groups were split into four breakout rooms each facilitated by one researcher from the steering group. Discussions in each room were themed according to a UK National Standard for Public Involvement and were clarified before opening up the discussion. All collaborators consented to recording of breakout discussions which were also annotated by scribes present at the time. Records and personal data from discussions were stored according to GDPR regulations (61). Notes were retrospectively checked for accuracy against recordings by the lead author.

### Data Analysis

Insights from the workshop were pooled and analyzed using conceptual mapping, a systematic process for delineating and identifying latent themes and patterns in large quantities of data (62). Once completed, results were formatted into a structure based on the NIHR Values and Principles Framework (49). Finally, all collaborators were invited to review the draft guidance and make further suggestions which were integrated and finalized by the steering group.

### Ethical Approval

The Imperial Research Governance and Integrity Team (RGIT) was consulted about all proposed activities prior to starting. As this study is classified as PPI, those in attendance are considered as collaborators not sources of data, and therefore no ethical approval is required.

### Reporting and Quality Control

The Guidance for Reporting Involvement of Patients and the Public (GRIPP) 2 checklist was used to help improve the design and reporting of PPI in this project (63). All instances of when and how public collaborators (non-psychedelic researchers) influenced the guidance were logged and discussed among steering group members. Quality checks on the final guidance document were performed using an modified framework (45) originally developed for the analysis of PPI strategies (Table 1). This draws on the 4Pi National Involvement Standards (64) to outline five key domains (principles, purpose, presence, process, and impact) and provides supporting questions against which PPI strategies can be evaluated. We modified questions to support our objectives which were not to develop strategy as such, but rather guide the planning of such strategies. Domains are rated as “unmet,” “partially met,” or “fully met” depending on how many criteria are met.

**Table 1:** Analysis framework based on 4Pi National Involvement Standards.

**4Pi domain**	**Definition used for analysis**	**Questions to support analysis**
Principles	• A set of values that inform meaningful involvement	1. Are values identified? e.g., “equality and diversity impact assessments inform our strategy”
		2. Is there evidence that values influence the strategy?
		3. Are principles stated?
Purpose	• Makes it explicit why people are involved	1. Specifies need for purpose or aim?
	• Describes why people are involved	2. Prompts objectives recorded?
	• Provides a rationale/goal for activity	
Presence	• Describes which groups/people need to be involved to shape and achieve the stated purpose	1. Who is the guideline author? 2. Who has influenced the guideline? 3. Recognizes specific target groups/populations?
Process	• Describes how involvement will happen	1. Does the guide facilitate plans to achieve the purpose or aim?
	• Sets out a series of relevant/appropriate methods or steps to achieve aim/objectives	2. Are time bound specific included? 3. Does the guideline support reporting mechanisms?
	• Indicates opportunity for reflection/learning/evolution over time	4. Is accountability addressed?
Impact	• Describes the difference involvement will make (intended/short-medium-long-term)	1. Is there evidence of success/impact criteria? 2. Are there defined mechanisms to assess impact? 3. Are there defined mechanisms for measurement and/or evaluation?

*Adapted from Matthews et al. (45)*.

## Results

Once organized by the steering group, the co-design workshop was attended by a total of 26 people, a breakdown of the groups in attendance is shown in Table 2. Workshop and breakout discussions ran without interruption from technical issues, and were captured on both video recording and in writing.

**Table 2:** Breakdown of groups attending a co-design workshop aimed developing guidance for PPI in psychedelic research.

**Collaborator key groups**	**Total**
Group facilitators	4
Scribes	5
Experienced PPI	4
Former participants of clinical psychedelic trial	4
Former participants of non-clinical psychedelic studies	5
Researchers linked to psychedelic research	4

*PPI, patient and public involvement*.

**Figure 2:**
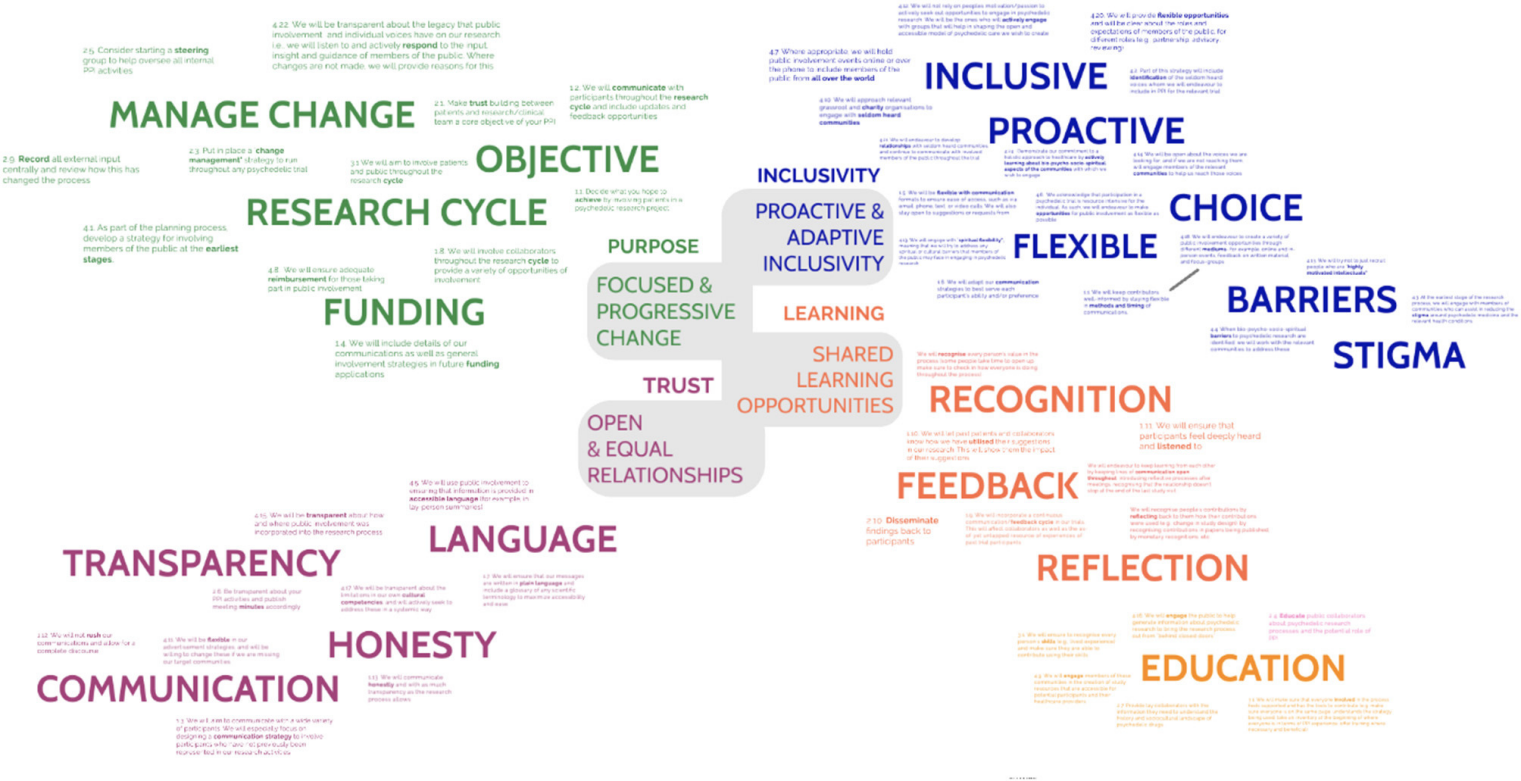
A scaled version of the conceptual map of content from the co-design workshop. Themes are grouped into four distinct but interrelated areas defined by core values of trust, purpose, inclusivity, and learning.

**Figure 3:**
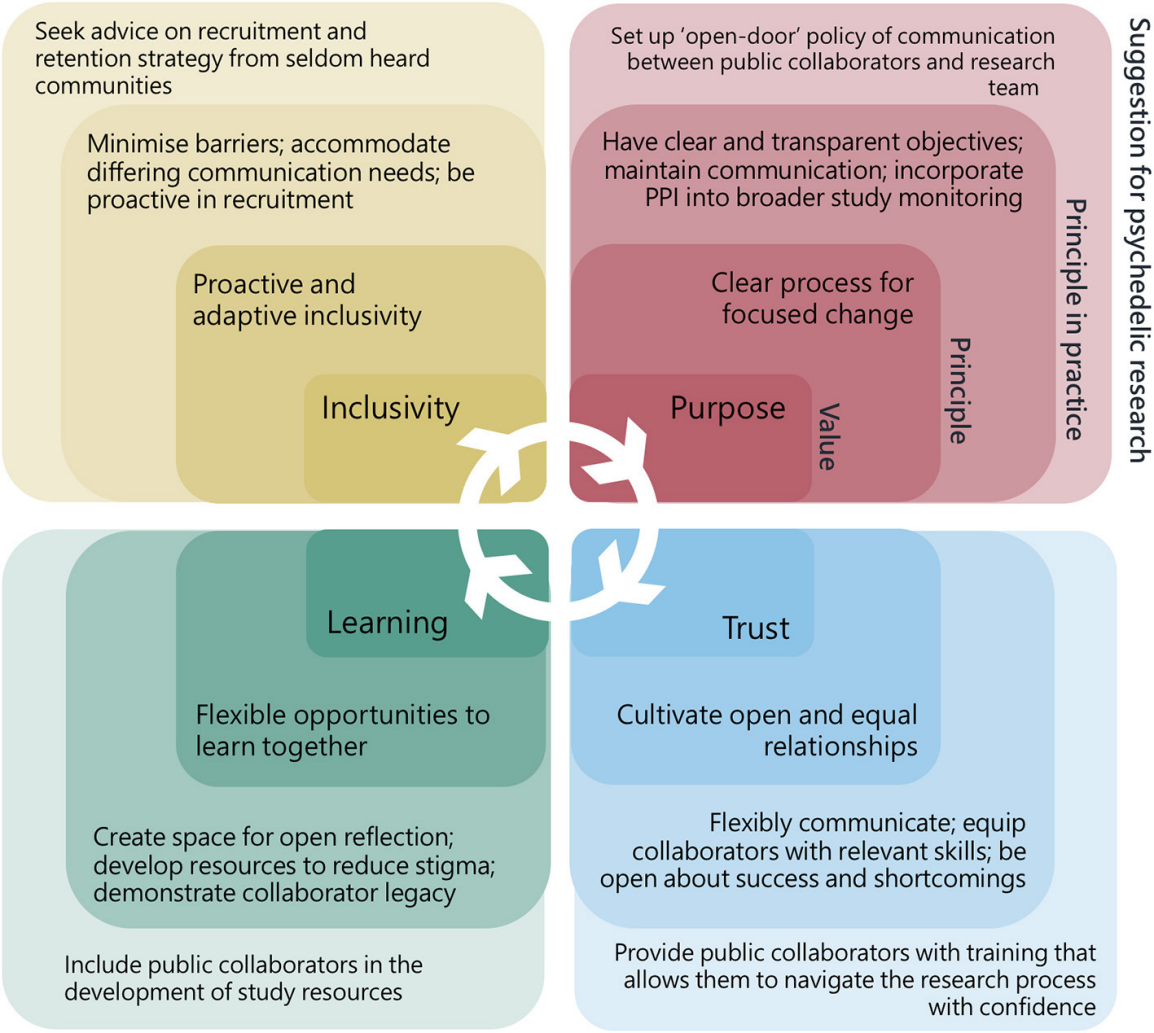
Values, principles, principles in practice, and suggestions for patient and public involvement in psychedelic research. Every value is relevant to all four areas of PPI and also bring more specific principles on which to build specific actions.

Concept mapping of the workshop is shown in Figure 2. Discussions exposed the main themes relevant to PPI in psychedelic research, these could be classified by four distinct but interrelating values and principles described below and illustrated in Figure 3. Suggestions for how these principles could be incorporated into the psychedelic research were arranged into the guidance document by steering group members.

### Inclusivity: Proactive and Adaptive Inclusion

Public collaborators highlighted the need for those working on research in psychedelics to “reach out” and be proactive in seeking patient and public contributors. Stigma and misunderstanding about psychedelic compounds, and in some cases, mental health, could act as barriers to involvement in more hesitant groups for whom the only point of reference for psychedelics might come from their community, laws or popular culture. Relying on a single PPI activity advert could therefore lead to a homogenous group of self-motivated individuals, lacking a diversity in opinions. Solutions to such problems included ways to “enable” involvement, for example, by being flexible and offering a choice of different means to access PPI opportunities (e.g., in person, by post, or online).

Two of the groups raised the importance of demonstrating open awareness and deliberate attempts to address inclusion in all areas of research, from participant recruitment, to engagement with the media, and other members of the core research team. If underrepresented groups are not coming forward, it was suggested that researchers could utilize PPI strategies to engage with these populations, for example, by going directly to community groups and grass-roots organizations to ask how to better connect with relevant groups on the topic of psychedelics.

### Learning: Flexible Opportunities to Learn Together

Those who had taken part in a psychedelic study all stressed the significance of their experience and desire to get involved in the research, but that they lacked opportunity to contribute further once they had ended their participation in a study. Several suggestions were raised around how former participants could contribute, such as through feedback forms or post-trial forums. It was observed how non-researchers could bring useful skills into the research team, for example some collaborators at the workshop drew on professional knowledge of project management and public relations when giving advice. Building spaces in which skills might be harnessed, recognized, and results fed-back would help contributors feel more valued.

Equally, the group of psychedelic researchers confirmed how much there was to learn from both former-participants and experienced public collaborators, especially in a field which is growing and both participants and researchers are significantly affected by outcomes. Both public contributors and researchers recognized the need to develop their own knowledge and skills before being able to fully contribute. Educational resources could be made available to cover a more detailed background of psychedelics or public involvement theory and could also be co-produced with specific populations in mind.

### Purpose: Clear Processes for Focused Change

Those psychedelic researchers outside the steering group had a limited understanding around the motives for undertaking PPI, recognizing that clear objectives would be an important part of any PPI strategy. A similar point was raised separately among the group experienced in PPI, who also pointed out the need to revisit and update objectives when implementing a strategy.

Suggestions for how PPI could influence psychedelic research changed with context, for example, impact might look very different in a psychedelic survey study to a clinical trial. There was an agreement amongst all discussion groups that some PPI outputs could be useful regardless of study specific context. A proposal was made that PPI activities adapt to include study specific populations if indicated. This might allow for more “change management” principles to feedforward into future studies and ensure PPI at the earliest stage of research design and funding applications.

Those from outside of research expressed a need for transparency around research activities, naming an individual accountable for PPI changes within the psychedelic research team was seen as a useful way to ensure any changes were actioned, and would give a clear point of contact for public contributors. In some cases, it might not be possible to be completely transparent, such as in trials where research design requires blinding conditions.

### Trust: Cultivate Open and Equal Relationships

Non-researcher discussion groups all expressed a need to be treated as equals by researchers. Feeling left out of the research process might be easily, if unintentionally, done through inaccessible language or deficient planning. This could be addressed by engaging with public collaborators while in the planning phase and when creating study documentation.

Additionally, there was a call for researchers to be open about both the positive and negative factors affecting psychedelic research such as specific political issues, resource limitations, and conflicts of interest. Honesty and humility were also seen as important personal qualities for engaging with public collaborators. Suggestions for how researchers could be more open include: circulating meeting minutes, incorporating PPI into all stages of the research cycle and including in funding decision-making. Ensuring trust is a value which guides all communications, through thoughtfully moderating the language.

### Impact of PPI on Guidance

External collaborators contributed to the development of this guidance at every point from conception to completion. Most material used to develop content originating from the workshop but some was used by the steering group, these additions were reviewed and approved by collaborators.

### Quality Control

When evaluated against the quality framework (Table 1), the guidance fully met the criteria of principles, purpose and presence domain. The “process” domain was partially met for not explicitly describing accountability lines. As the guidance is not explicit about mechanisms to assess impact and evidence for success criteria, the impact domain was also partially met.

## Discussion

Guidance for creating a patient and public involvement strategy in psychedelic research were developed by researchers and patient and public collaborators. Using co-design methods, we identified fundamentals to PPI in psychedelic research and built these into a guidance document to help inform psychedelic research teams and their collaborators. Four key values: purpose, inclusivity, learning, and trust emerged as central to PPI in psychedelic research.

To our knowledge there is no published guidance which seeks to address PPI in the field of psychedelic research. At a fundamental level, our document holds a number of parallels with existing PPI guidance (49, 64) including the UK National Standards for Public Involvement upon which our workshop was based (8). This is likely a reflection of the common premise of PPI, which is grounded in ideals of equity and democracy. Significantly, the majority of content is original and distinct from generalized as it places more emphasis on the fundamental importance of building trusting relationships with public collaborators in research specific to psychedelics. Trust has long been placed at the center of guidance related to psychedelic research (65–69) in which participants are encouraged to “trust, open, and let go” (70). In this context, the responsibility lies with the researcher to be trustworthy, rather than a participant to be trusting (71). We propose that psychedelic research built on open and trusting PPI will result in superior outcomes and experiences for participants. The vocabulary used when developing participant information, outcome measures and interventions for psychedelic research might feel alienating or less socially acceptable to some audiences (72). Seeking the advice of others can help build empathy and help researchers to understand the fears and beliefs of potential participants of psychedelic research. This is particularly important, as researchers often knowingly or unknowingly act in a guiding capacity for participants, and can consequently influence expectancy bias (73).

Our guidelines promote a proactive approach to inclusivity, by prompting researchers to make an active effort to ensure opportunities for engagement are given to a diverse audience. Similarly, “positive action,” a principle relating to employment practices, aims to improve workforce diversity by guiding how candidates are fairly recruited and promoted (60). For many, there is an assumption that with higher ethical standards, contemporary psychedelic research will not follow the same path of discrimination, cultural appropriation (43) and other abuses of power seen in early psychedelic studies (74, 75). However, over 80% of participants in clinical trials are White (38), suggesting that more work has to be done to re-balance this representation. In addition, when conducted in naturalistic settings, psychedelic research risks amounting to cultural appropriation if indigenous communities are not consulted (43). Our guidance recognizes that making psychedelic research accessible requires more than an “open door” recruitment policy. Although unintentional, a passive approach can lead to a perpetuation of the problem of inequality in a field less accessible to minority populations (37) whom are already more inclined to mistrust medical and research institutions (76). A good example of positive actions are described by Williams et al. (42) who use culturally informed research design to help participants from minority backgrounds relate and engage with research into MDMA assisted therapy. Cultural competency is an essential attribute for psychedelic researchers and clinicians (77), we hope the guidance presented here will prompt researchers to take similar purposeful actions, to address gender, socioeconomic, educational and age disparities in psychedelic research (78).

Participating in a psychedelic trial demands great courage, and is often reported as one the most meaningful experiences of a lifetime (51). Creating a space in which contributions can be acknowledged after the completion of participation in the psychedelic study emerged as a priority in the guidance. Our guidance recognizes research as an iterative process (79); for researchers only seeking answers to specific research questions a great opportunity for mutual learning can be lost in failing to engage participants beyond the end of a psychedelic trial. Collaboration with former-participants could feedforward to strengthen future research, especially when knowledge is managed and shared across trials with this specific purpose. Such transparent PPI processes have the potential to counter threats to the legitimacy and potential of the *psychedelic renaissance* (1).

Psychedelic researchers face a great task in conducting high quality research. Besides the numerous hurdles of procuring heavily regulated drugs, researchers are expected to balance participant welfare with a need to complete trials in a timely and cost-effective manner. As our guidance shows, carrying out good PPI in psychedelics is not straightforward, and as it stands PPI is rarely included as part of undergraduate training for researchers. Subsequently, researchers must seek funding, experience and skills for PPI under their own volition. Greater education and resources for PPI would not only be beneficial to the field of psychedelic research, but medical research more broadly.

There are a number of limitations to our guidelines and the methods used to develop them. At the time of publication, the guidelines were yet to be tested over the course of a whole research cycle, and may need to be adapting accordingly (45). Furthermore, this rapidly evolving field of research makes it difficult to capture the impact of PPI activities. Future studies in this area could seek to evaluate the effects of PPI strategies and the guidance upon which they are based.

## Conclusion

One of the appeals of psychedelics shared by researchers and participants alike is the promise of discovery. The quality of present-day psychedelic research means all stakeholders are on the same journey into unknown territory. For many, there is an assumption that with higher ethical standards, contemporary psychedelic research will not follow the same path of discrimination and other abuses of power seen in early psychedelic studies. We hope that the guidelines presented here will assist in greater incorporation of the voices of the wider community in psychedelic research, and that it will continue to develop alongside this exciting field of research. PPI when carried out systematically and thoughtfully, offers a means of creating more valuable psychedelic research through directly addressing the complex power dynamic between research team and the public at large.

## Data Availability Statement

The raw data supporting the conclusions of this article will be made available by the authors, without undue reservation.

## Author Contributions

JC conceptualized the study, acted as lead author and main point of contact for collaborators. JB and MS contributed to the initial design of the study. JB, JC, LL, MP, MS, and SJ oversaw the design, planning and running of the workshop, and contributed to processing the proceedings. JC wrote the first draft. JB, JC, LL, RC-H, MP, MS, and SJ reviewed the manuscript prior to submission. All authors contributed to the article and approved the submitted versions.

## Funding

Funding for this project was provided by the Imperial College London, Centre for Psychedelic Research.

## Conflict of Interest

The authors declare that the research was conducted in the absence of any commercial or financial relationships that could be construed as a potential conflict of interest.

## Publisher's Note

All claims expressed in this article are solely those of the authors and do not necessarily represent those of their affiliated organizations, or those of the publisher, the editors and the reviewers. Any product that may be evaluated in this article, or claim that may be made by its manufacturer, is not guaranteed or endorsed by the publisher.
